# Sensitivity analysis of bi-metal stacked-gate-oxide hetero-juncture tunnel fet with Si_0.6_Ge_0.4_ source biosensor considering non-ideal factors

**DOI:** 10.1371/journal.pone.0301479

**Published:** 2024-06-11

**Authors:** Rittik Ghosh, Rajeev Pankaj Nelapati, Priyanka Saha, Ravikumar Chinthaginjala, Tai-hoon Kim, Kumar S.

**Affiliations:** 1 School of Electronics Engineering, Vellore Institute of Technology, Vellore, Tamil Nadu, India; 2 Department of Electronics and Communication Engineering, C.V Raman Global University, Bhubaneswar, India; 3 School of Electrical and Computer Engineering, Yeosu Campus, Chonnam National University, Yeosu-si, Jeollanam-do, Republic of Korea; 4 Data Science Research Laboratory, BlueCrest University, Monrovia, Liberia; Dayalbagh Educational Institute (Deemed University), INDIA

## Abstract

This article provides insights in designing a dielectrically modulated biosensor by adopting high-k stacked gate oxide proposition in a bi-metal hetero-juncture Tunnel Field Effect Transistor (BM-SO-HTFET) with Si0.6Ge0.4 source. The integrated effect of heterojunction and stacked gate oxide leads to enhanced electrical performance of the proposed device in terms of carrier mobility and suppressed leakage current. Nano-cavity engraved beneath the bi-metal gate structure across the source/channel end acts the binding site of the biomolecules to be detected. This Configuration leads to improved control of biomolecules over source/channel tunnelling rate and the same is reflected in the sensing ability of the device while extracting the ON current sensitivity (SON) of the sensor. The reported biosensor is simulated using Silvaco ATLAS calibrated simulation framework. The analysis of the device sensitivity is carried out varying dielectric constants (k) of various biomolecules, both neutral as well as charged. Our study reveals that BM-SO-HTFET with Ge mole fraction composition x = 0.4 exhibits sensitivity as high as 4.1 × 10^10^ for neutral biomolecules and 3.2 × 10^11^ for positively charged biomolecules with k = 12. Furthermore, a transient response profile for the drain current with various biomolecules is explored to determine the varying settling time. From the simulation results, it is noted that BM-SO-HTFET exhibits ON current sensitivity of 4.1 × 10^10^ and 3.2 × 10^11^ for neutral and charged biomolecules respectively. In addition to this, for highly sensitive and real time detection of biomolecules, the impact of temperature and certain non-ideal factors drifting from ideal case of fully filled cavity have also been considered to analyze its optimum sensing performance.

## 1. Introduction

Present day microbiological progress has steered the researchers to a focused and streamlined path in biosensing performance investigations. Biological molecules or biomolecules or bio-species are fundamental ecological components. DNA (Deoxyribonucleic acid), Biotin, Streptavidin, Gluten, Zenin, Keratin, Gelatin play critical roles in lifeforms. Field Effect Transistors (FET) based lab-on-chip biosensors for efficient and accurate label free detection of biomolecules have emerged as a major interest in the research arena for both ideal and non-ideal issues [1,2]. The complex lab based expensive sensors can be substituted by FET based biosensors which have easy fabrication steps, higher sensitivity, higher lifetime, high reliability, low power consumption and low cost [3]. To obtain the best performance of a FET based biosensor it should be highly sensitive. Ion sensing FET (ISFET) had become a prime focus for researchers in 1970s for sensing biomolecules with charge density [4]. But as reported in [5], ISFET could not detect neutral biomolecules. The above problem has been solved with the introduction of dielectrically modulated FET based biosensors with the potential of sensing neutral biomolecules as well as biomolecules with charge density. In dielectrically modulated FET based biosensors, nanogap engineering is implemented under the gate metal by wet etching the gate oxide [6]. The biomolecules are selectively captured with the help of receptors in the engineered nanogap. With the applied gate bias, the effectual gate-channel capacitance varies with different dielectric constant values of different biomolecules localized inside the engineered nanogap giving rise to readable signals in the form of drain characteristics, transfer characteristics, channel conductance, threshold voltage characteristics. The dielectric constant values vary with varying biomolecules such as Streptavidin, Biotin, 3-Aminopropyl Triethoxysilane (APTES), Gluten, Zenin, Keratin, Gelatin having k = 2.1, 2.63, 3.57, 5, 7, 10, 12 respectively [7]. The integration of dielectrically modulated n-FET and p-FET based biosensors with Complementary Metal Oxide Semiconductor (CMOS) architecture provides satisfactory compatibility for further logic applications. Bulk Metal Oxide Semiconductor Field Effect Transistor (MOSFET) emerged as a suitable candidate for dielectrically modulated biosensing [8]. But in accordance with Moore’s Law, the downscaling of technology nodes causes MOSFETs to pose several problems. It’s limitation to 60 mV/dec Subthreshold Swing (SS) at ambient conditions, giving rise to Short Channel Effects (SCEs), KT/q limit, increase in leakage current, high static power consumption makes MOSFET based biosensors not suitable for efficient sensing of biomolecules as well as long response time [9,10]. Dielectrically modulated Tunnel Field Effect transistors (DM-TFETs) based biosensors in recent times are under extensive research due to their capability of overcoming the above-mentioned limitations with MOSFET based biosensors [11].

TFET is also ideal for low power Very Large-Scale Integration (VLSI) utilities because of their significantly low OFF current therefore having reduced static power consumption [12]. DM-TFET based biosensors provides sub-60 mV/dec SS and faster response time. The Band-to-Band Tunnelling (BTBT) phenomenon of TFET based biosensors makes them essentially important candidates for biosensing operations with dielectric modulation approach [13,14]. Recently, Covid-19 illness causing virus, SARS-CoV-2 has come under tremendous attention due be-cause of the pandemic break-out that has taken countless lives and continues as a threat and demands fast and accurate detection [15]. It is reported in [3] that SARS-CoV-2 virus can be detected by TFET based biosensors. Besides the advantages of TFET based biosensors over MOSFETs, TFETs have extremely low ON current (I_ON_) and ambipolar behaviour for conductivity which can lead to poor performance and even circuit failure [16]. Researchers are working to find out the best possible architecture that can increase the I_ON_ as well as reduce the ambipolar current behaviour. As reported in [17], asymmetric doping profiles of source and drain can suppress ambipolar current behaviour and the I_ON_ can be improved by sharp source doping, thin silicon body and a double gate architecture that will improve the gate controllability on the channel charge. The Z-shaped gate hetero dielectric horizontal source pocket TFET (ZHP-DM-TFET) based biosensor [18], Gate All Around TFET (GAA-TFET) [19], SiGe Source and Pocket-Doped Channel [1], N+ Pocket Doped Vertical TFET Based Dielectric-Modulated Bio-sensor [20], Dielectric Modulated Dual Channel Trench Gate TFET-Based Bio-sensor [21], Back-Gate Bias and Front-Gate Engineering DMTFET-Based Bio-sensors [22] focuses on improving the ON current sensitivity by improving the carrier mobility. Lately, tunnel FETs with integrated hetero-juncture architecture have grabbed attention [23]. The mindful selection of bandgap modulating materials at the source side allows improvement in ON-state current as well as the sub-threshold slope (SS) by improving the tunnelling rate. Therefore, in terms of considering ON-state current and SS as a sensitivity parameter, hetero-juncture architecture in TFETs is of real advantage. However, hetero-juncture architecture also leads to an increase in the sub-threshold current which is an issue in terms of power consumption [24]. Therefore, further research is needed to improve the hetero-juncture architecture in TFETs.

In this work, a Bi-Metal Stacked-oxide Hetero-juncture Tunnel Field Effect Transistor (BM-SO-HTFET) dielectrically modulated biosensor device has been utilized with an incorporated Si_0.6_Ge_0.4_ source. The engineered gate oxide architecture facilitates the carrier mobility by mitigating the leakage current. Bi-gate metal structure improves tunnelling at the source-channel interface thereby improving the ON current and higher control over the channel charge [14]. This delves deeper into the sensing abilities of the BM-SO-HTFET dielectrically modulated biosensor with various biomolecules consisting of dielectric constant values ranging from 5 to 12. The biosensor device exhibits ON current sensitivity for neutral biomolecules (SON = 4.1 × 10^10^ at k = 12), charged biomolecules (SON = 3.2 × 10^11^ at k = 12) for fully filled nanogap at ambient temperature. Furthermore, real-time bio-sensing performance considering non-ideal nanogap filling is extensively investigated in terms of ON current sensitivity.

### 1.1. Device architecture and simulation framework

**Fig 1:**
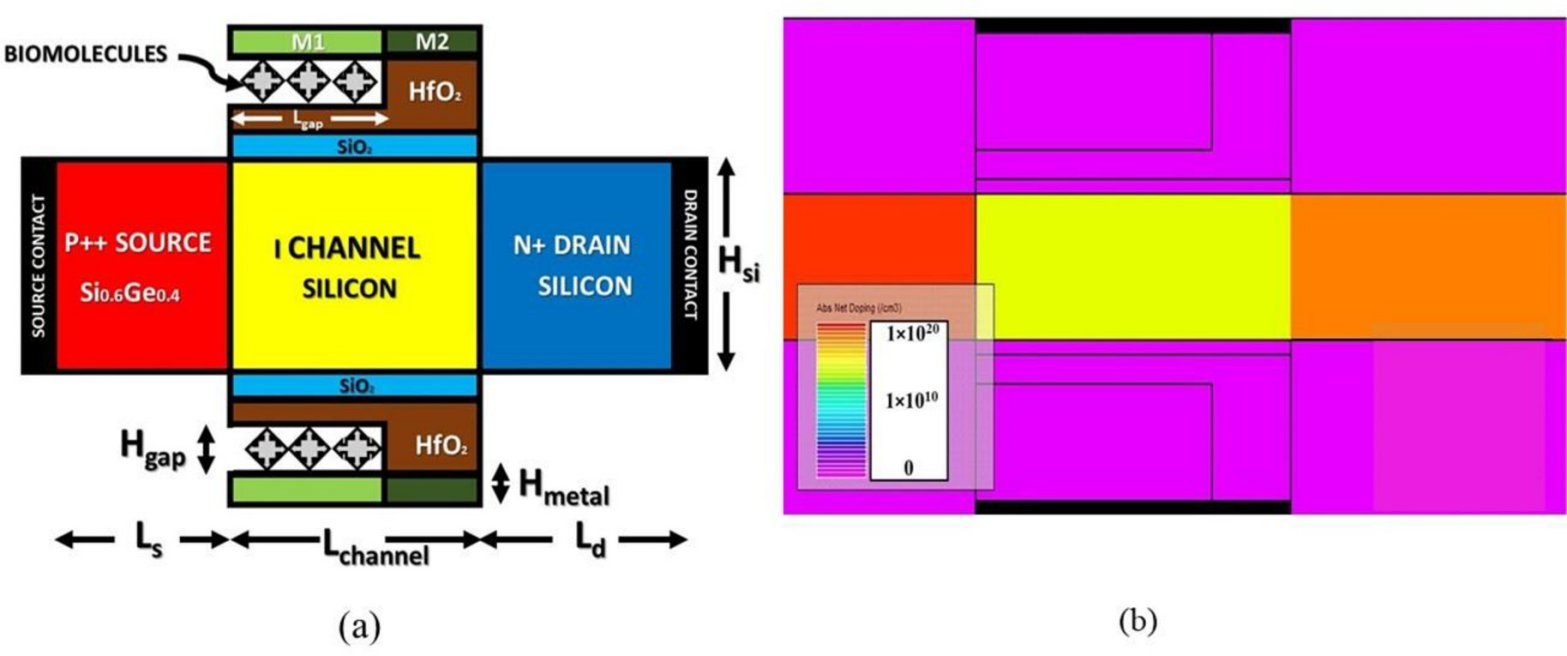
(a) 2D illustrative (b) TCAD Contour doping profile of BM-SO-HTFET biosensor with Si_0.6_Ge_0.4_ source.

Fig 1(A) presents the 2-D illustration of BM-SO-HTFET dielectrically modulated biosensor. Si_0.6_Ge_0.4_ has been incorporated in the source with a highly P-type doping profile over the mildly (intrinsic) doped silicon channel forming a hetero juncture. A 40 nm channel length is considered. The SiO_2_ (low-κ insulator) depth is 1 nm whereas HfO_2_ depth is 2 nm. A nanogap of 30 nm distance and body depth of 10 nm is considered. The Si0.6Ge0.4 source is 1 × 10^20^ cm-3 doped; silicon drain is 1 × 10^18^ cm-3 doped. The silicon channel is 1 × 10^15^ cm-3 lightly doped shown in simulated contour doping profile in Fig 1(B). The simulated BM-SO-HTFET has a high-k gate stack that mitigates the leakage current subsequently improving I_ON_, while also being thermodynamically stable [25,26]. Bi-metal with work-function of tunnelling gate, ϕ_tg_ = 3.8eV and work-function of auxiliary gate, ϕ_ax_ = 4.8eV is utilized for constructing the gate architecture [2]. To emulate biosensing, the nanogap is considered air-padded at k = 1. At air-padded nanogap (k = 1), the effective gate-channel capacitance becomes less influential as compared to biomolecules padded nanogap thereby mitigating effective tunnelling. Therefore, a metal with lesser work-function (ϕ_tg_ = 3.8 eV) positioned over the nanogap to overcome this is-sue. It is known that a filled nanogap will enhance the tunnelling vertical electric field leading to acute band bending and increased tunnelling. Metal with increased work-function (ϕ_ax_ = 4.8 eV) utilized over high-κ insulator, HfO_2_ to accurately facilitate charge carrier tunnelling for padded nanogap. Therefore, tunnelling gate controls subthreshold swing (SS) while auxiliary gate governs the leakage current [2]. From reported articles it is known that mono nanogap biosensors exhibit improved outputs in comparison to mirrored nanogap biosensors due to their ability to impregnate acute band-bending at the tunnelling interface for various biomolecules simultaneously providing improved coverage area of the nanogap. The split nanogap architecture in mirrored nanogap devices reduces the coverage area of the nanogap thereby degrading effective gate to channel capacitance [2].

**Fig 2:**
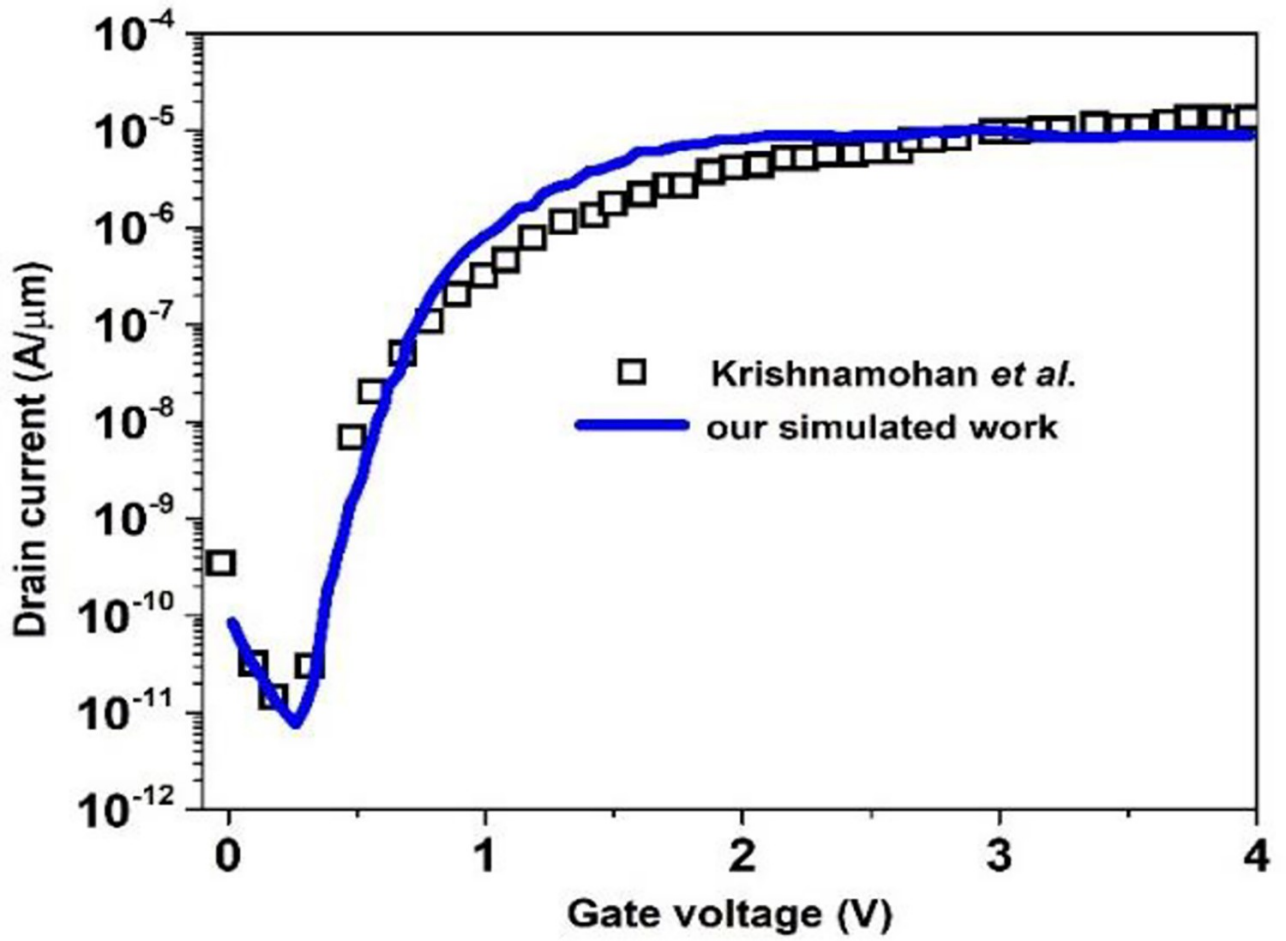
Calibration of transfer characteristics of SiGe PIN TFET [26].

BM-SO-HTFET biosensor measurements are presented in Table 1. The simulation of the BM-SO-HTFET is carried out with SILVACO ATLAS commercial TCAD tool [27]. The Wentzel-Kramer-Brillouin (WKB) approximation based non-local BTBT model has been implemented to incorporate effective tunnelling due to band-bending at the tunnelling juncture. CONMOB and FLDMOB models are used for mobility degradation. Furthermore, the classical Drift Diffusion model along with Shockley-Read-Hall (SRH) model and Auger model has been incorporated for facilitation of carrier generation and recombination. The Band Gap Nar-rowing model (BGN) and Fermi-Dirac statistics were further included to procure accurate band-to-band tunnelling. The calibration of the mole fraction parameter (x) has been done with the experimental results reported in [28] and implemented in the SILVACO ATLAS TCAD tool. The experimental results of the device transfer characteristics of SiGe PIN TFET [29] are fitted with the simulation results in terms of physical models and device specifications. The calibration results are highlighted in Fig 2.

**Table 1:** Measurements of the simulated BM-SO-HTFET biosensor.

S. No.	FET specifications	Measurements
1.	Channel length (L_channel_)	40 nm
2.	Silicon channel depth (H_si_)	10 nm
3.	Source length (L_s_)	25 nm
4	Drain length (L_d_)	35nm
5	Nano-gap length (L_gap_)	30nm
6	Nano-gap depth (H_gap_)	8 nm
7	Gate metal depth (Hmetal)	1nm
8	Low-k (SiO2) and High-κ (HfO2) dielectric depth	1nm and 2nm respectively
9	High-k (HfO2) dielectric depth below M_2_	nm

## 2. Simulation results and discussions

**Fig 3:**
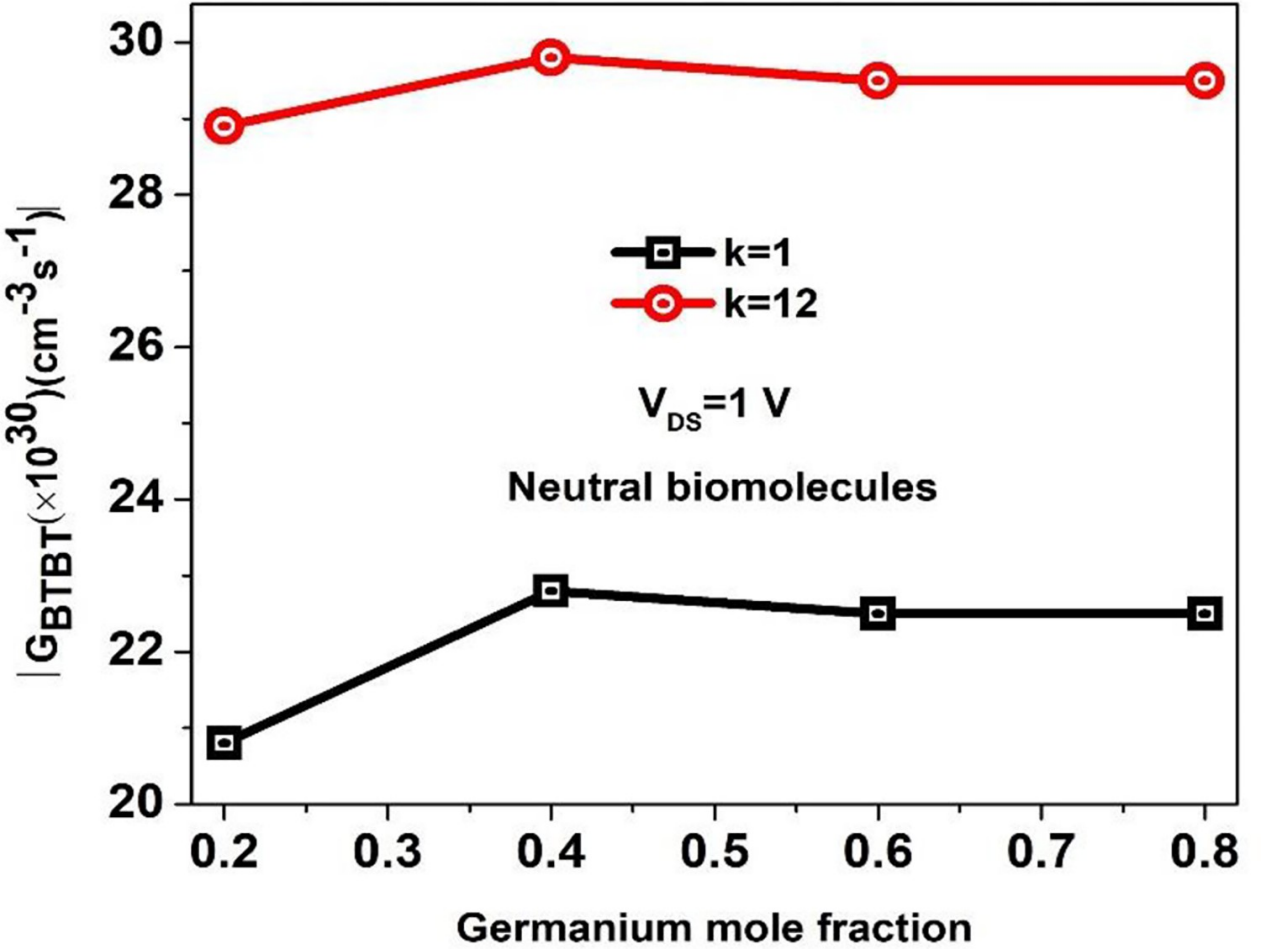
Band to Band tunnelling rate for empty and filled nanogap for different Ge mole fraction values of BM-SO-HTFET.

A Si_1-x_Ge_x_, a bandgap tuneable alloy semiconductor, is incorporated in the P^++^ type source of BM-SO-HTFET dielectrically modulated biosensor. The mole fraction composition of Ge is determined by mole fraction (x) values. From Fig 3, it is noted that as the value of x increases, it is observed that the bandgap of Si_1-x_Ge_x_ narrows till x = 0.4 for both empty and filled nanogap, thereby boosting the band-to-band tunnelling rate, GBTBT [29]. However, the GBTBT doesn’t improve at x = 0.6 and x = 0.8 values thereby making x = 0.4 a suitable choice for the simulation of BM-SO-HTFET.

**Fig 4:**
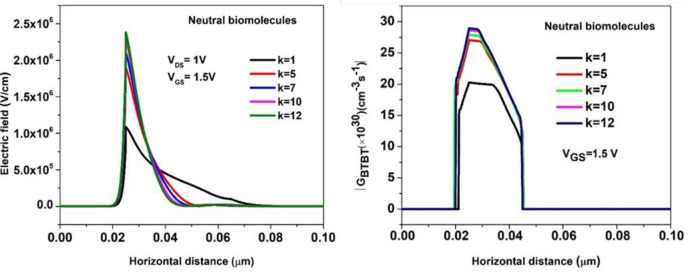
(a) Electric field profile (b) BTBT generation rate of the BM-SO-HTFET with increasing dielectric constant (k) values of the biomolecules.

Fig 4A highlights increment of the tunnelling vertical electrical field profile. It is observed that vertical electric field is greatest at k = 12 (2.4 × 106 V/cm) that can be attributed by the increased gate coupling effect. Fig 4B represents that the Band-to-band tunnelling generation rate (GBTBT) that increments at greater k values (28 × 1030 cm-3s-1 at k = 12). The non-local Band-to-band tunnelling model activates the BTBT dynamics at the tunnelling juncture of the biosensor device [30].

**Fig 5:**
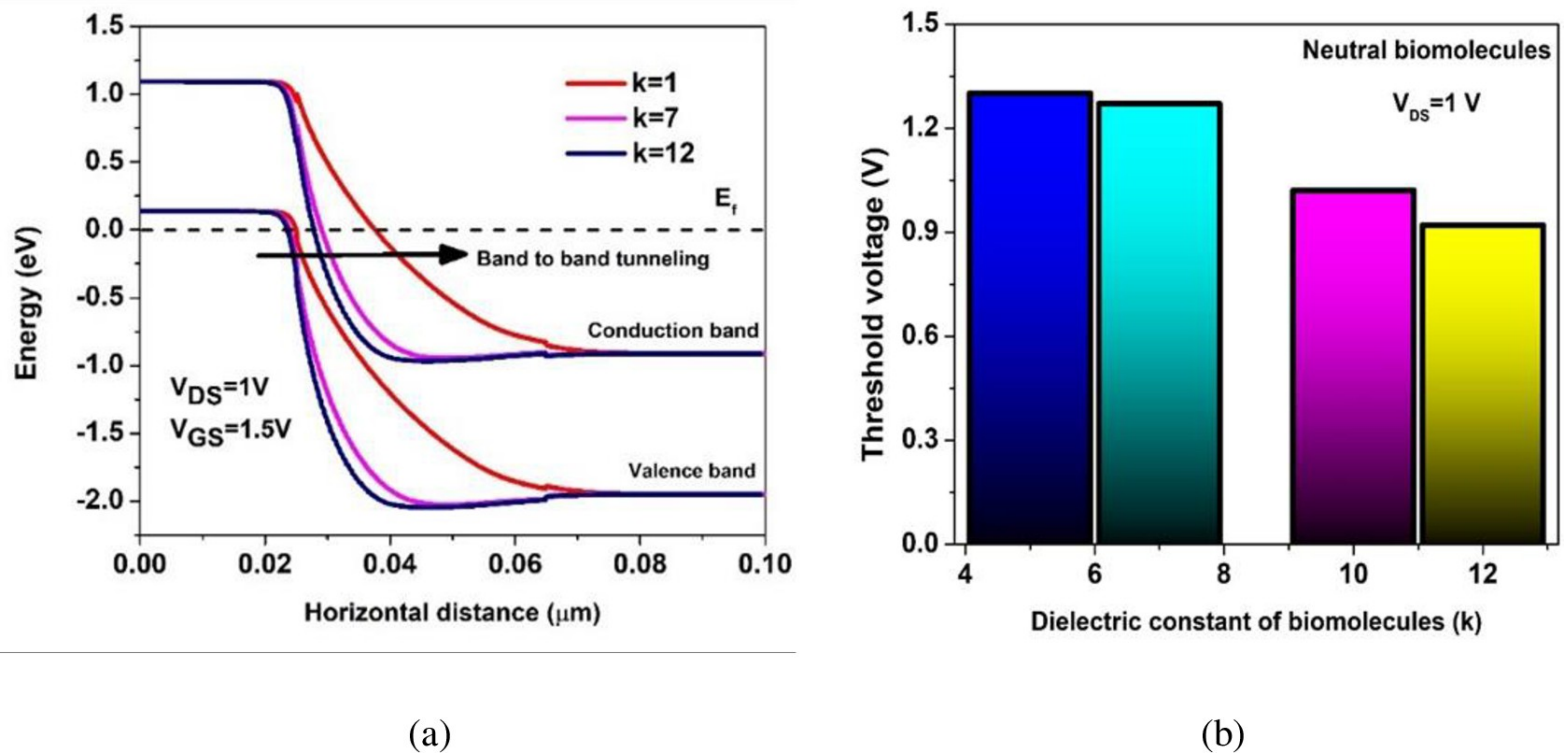
(a) Energy band diagram profile (b) Threshold voltage profile of the BM-SO-HTFET with increasing dielectric constant (k) values of the biomolecules.


The Energy Band diagram profile shown in Fig 5A highlights the acute band-bending at the tunnelling interface for increasing dielectric constants of the biomolecules that is mainly attributed by the ϕ_tg_ whereas, OFF current (I_OFF_) is governed by ϕ_ax_. Thereby, enhancing the BTBT generation rate (G_BTBT_) resulting in enhanced Ion values. Fig 5B represents the biosensor device threshold voltage profile for increasing dielectric constants of the biomolecules. As observed from the energy band diagram profile that the band gap narrowing with increasing dielectric constant of the biomolecules leads to increased vertical electric field thereby increasing ON current resulting in the decreased threshold voltage at greater dielectric constants.

$$G_{BTBT}=CE^{\sigma }exp\;\left(\frac{-B}{E}\right)$$ (1)

Here, C is a factor which is a function of electron effective mass, B representing tunnelling possibilities. Transitsion constant is represented by σ. Electric field is represented by E. The tunnelling possibility in tunnel FET devices are mainly governed by the Wentzel-Kramer-Brillouin approximation (WKB) [21].


$$T_{WKB}\approx exp\left(\frac{-4\lambda \sqrt{2m^{*}}\sqrt{E_{g}^{3}}}{3qђ(E_{g}+\Delta \phi )}\right)$$ (2)

Here, λ and m* represents the screening tunnelling span and the electron effective mass respectively. The semiconductor forbidden gap (bandgap) is represented by E_g_, △ϕ represents the tunnelling range in terms of energy. From (1), it can be noticed that the acute band-bending can be achieved with increased electric field. From (2), it is deduced that as the k values rise, the tunnelling span narrows and T_WKB_ surges thereby increasing drive current/ON current [30].

**Fig 6:**
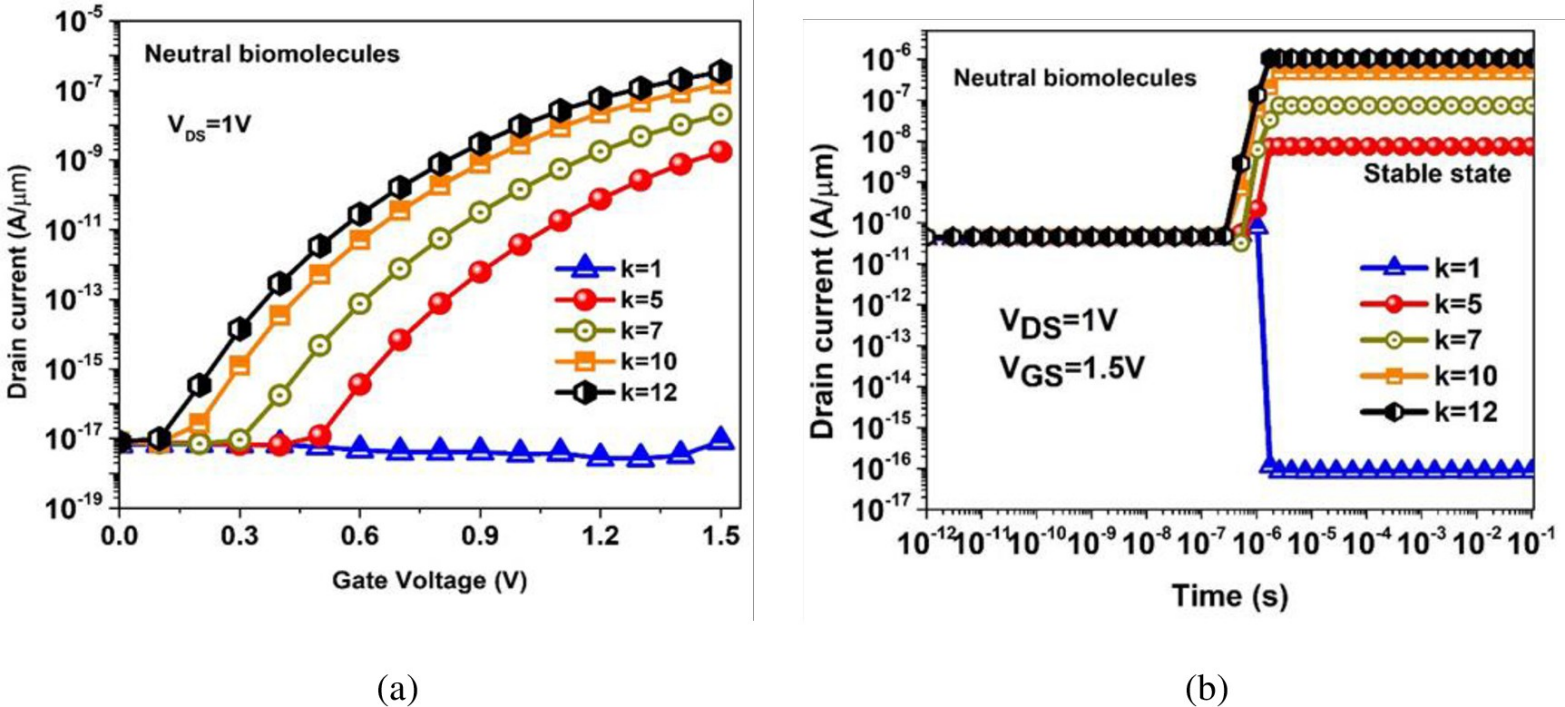
(a) DC Transfer characteristics (b) Transient response profile of the BM-SO-HTFET with increasing dielectric constant (k) values of the neutral biomolecules.

Fig 6A demonstrates the device transfer characteristics (I_DS_-V_GS_) of the BM-SO-HTFET for increasing dielectric constant (k) values of the localized biomolecules in the nanogap. It is noted that air-filled nanogap yields poor I_ON_ ~ 10^−17^ A/μm [31]. However, I_ON_ increases significantly with rising k values whereas maintaining an appreciably low I_OFF_ which improves ON-OFF switching. SNR, signal-noise-ratio (SNR) gets hindered in real time for these FET based biosensors [32]. Low I_OFF_ current results in reduced consumption of static power in traditional Bi-Metal TFET biosensor. Furthermore, researchers are implementing a transient response approach for exploiting the settling time of I_ON_ with different biomolecules inside the nanogap [32]. Biosensing parameters like sensitivity and selectivity for a certain applied gate voltage can be meticulously studied using transient response of a FET based biosensor device. Fig 6B shows the transient response of BM-SO-HTFET biosensor localizing different biomolecules inside the nanogap. It is evident from the analysis that as the k values for different biomolecules inside the nanogap increase, the I_ON_ increases and reaches a stable state at different settling times [30]. In the case of gelatin biomolecule (k = 12), I_ON_ takes the least settling time to reach transient stable state since vertical electric field induces acute band bending at the tunnelling junction due to higher dielectric constant of the biomolecule [30]. Whereas, for an empty nanogap (k = 1), prolonged settling time is observed to reach transient stable state as air-filled nanogap mitigates band bending at the tunnelling juncture, thereby reducing BTBT rate.

### 2.1. Sensitivity of ON current (S_ON_)


$$S_{ON}=\left|\frac{I_{ON}(biomolecules)}{I_{ON}(air)}\right|S_{ON}=\left|\frac{I_{ON}(biomolecules)}{I_{ON}(air)}\right|$$ (3)

The sensitivity of the biosensor is one of its most important metrics to determine the biosensing functionality. Various sensitivity profiles like threshold voltage sensitivity, I_ON_/I_OFF_ sensitivity, ON current sensitivity can be considered to analyse the performance of a biosensor. However, in this work, we consider sensitivity of ON current for BM-SO-HTFET biosensor as shown in (3). S_ON_ is defined by the fraction of ON current when the biomolecules are localized and absent in the nanogap. I_ON_ has been selectively considered to determine sensing performance since in TFETs I_ON_ depends on BTBT rate. BTBT however, is independent of temperature. Subthreshold Swing (SS), threshold voltage shift (△Vth) and I_ON_/I_OFF_ ratio depend heavily on thermionic emission phenomenon [2]. Hence temperature dependence of these parameters might hamper the sensing functionality of the biosensor. The dependence of sensitivity on temperature is elaborated under section 3.2.

**Fig 7:**
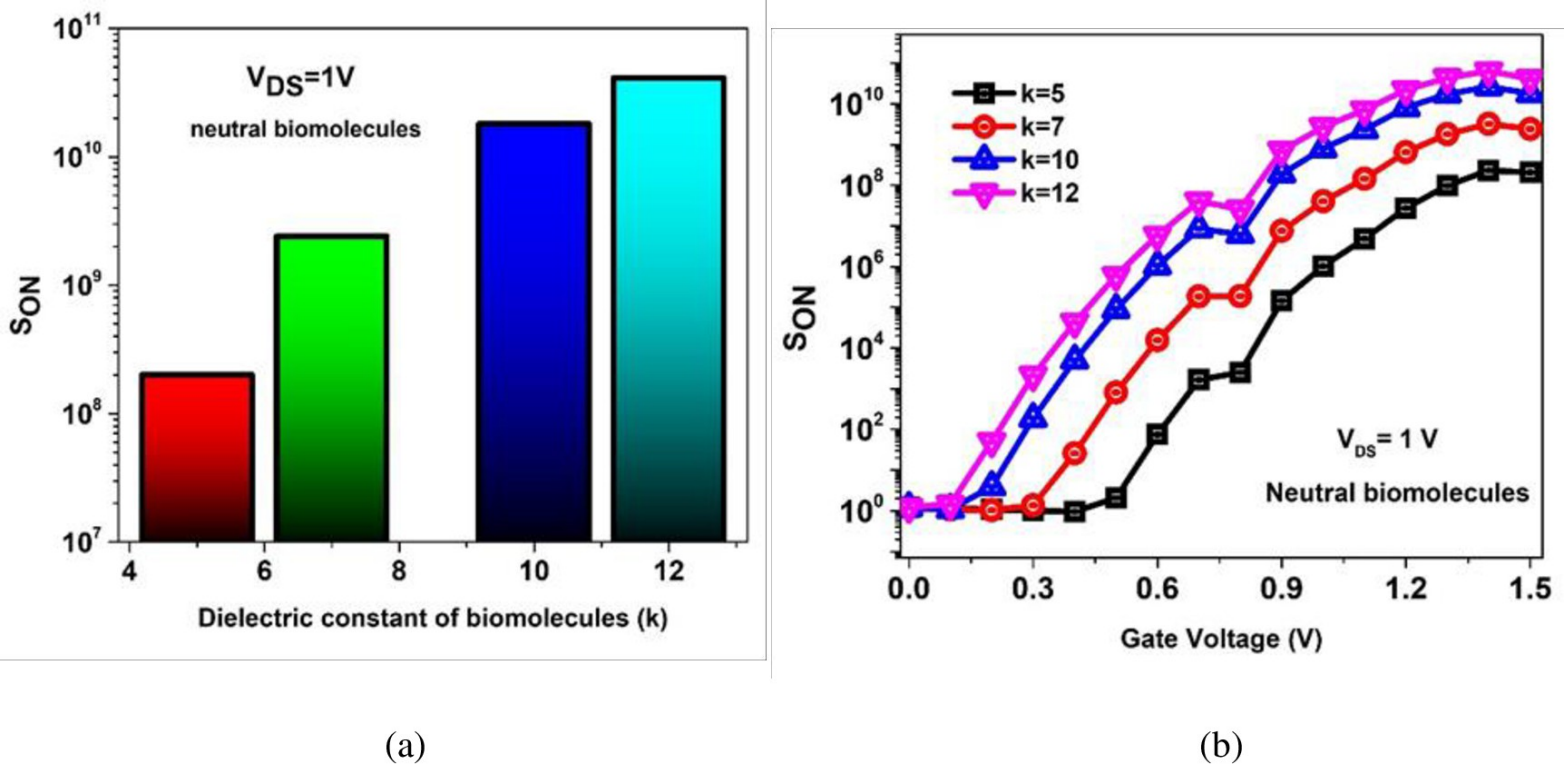
ON current sensitivity for (a) with increasing dielectric constant (k) values (b) gate voltage of the BM-SO-HTFET of the neutral biomolecules.

Asymmetrical doping of source (1 × 10^20^ cm^-3^) and drain (1 × 10^18^ cm^-3^) achieves better sensitivity of the TFET based biosensors [33]. Fig 7A highlights steep rise of S_ON_ for increasing k values and it can be noted the k = 12, gelatin yields the maximum sensitivity, S_ON_ = 4.1 × 10^10^. Fig 7B highlights the S_ON_ profile as a function of gate voltage. It is observed that peak S_ON_ (6.4 × 10^10^ at k = 12) for increasing dielectric constant of biomolecules is achieved at ~1.35 V gate voltage.

## 2.2. Impact of temperature on S_ON_

**Fig 8:**
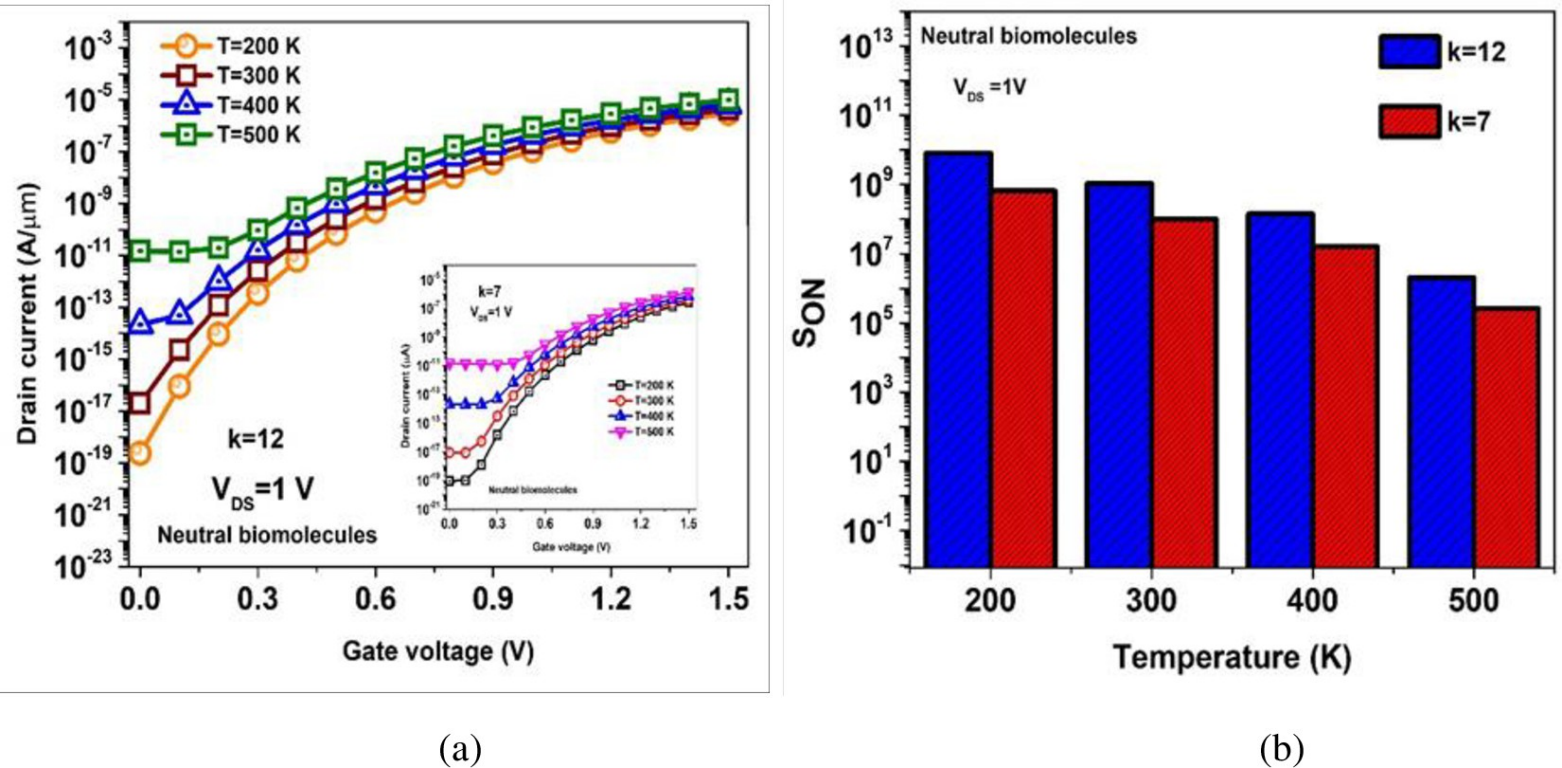
Drain current variation (b) ON current sensitivity (S_ON_) of the proposed BM-SO-HTFET with different temperature at k = 7, k = 12.

The sensitivity profile of dielectrically modulated biosensors can be furthermore investigated considering its variation from ambient temperature. It is evident from Fig 8A that I_OFF_ increases with increase in temperature as recombination rate increases in BM-SO-HTFET biosensor for k = 7 and k = 12 [34]. Insignificant change in I_ON_ at higher gate voltages is observed. Sensitivity varies with gate voltage and temperature. The I_OFF_ keeps on increasing in the presence of air in the nanogap with varying temperature as a result the ON current sensitivity (S_ON_) monotonically reduces as temperature rises, same can be observed in Fig 8B for biomolecules with dielectric constant (k = 7 &12).

### 2.3. Impact of charge biomolecules on S_ON_

**Fig 9:**
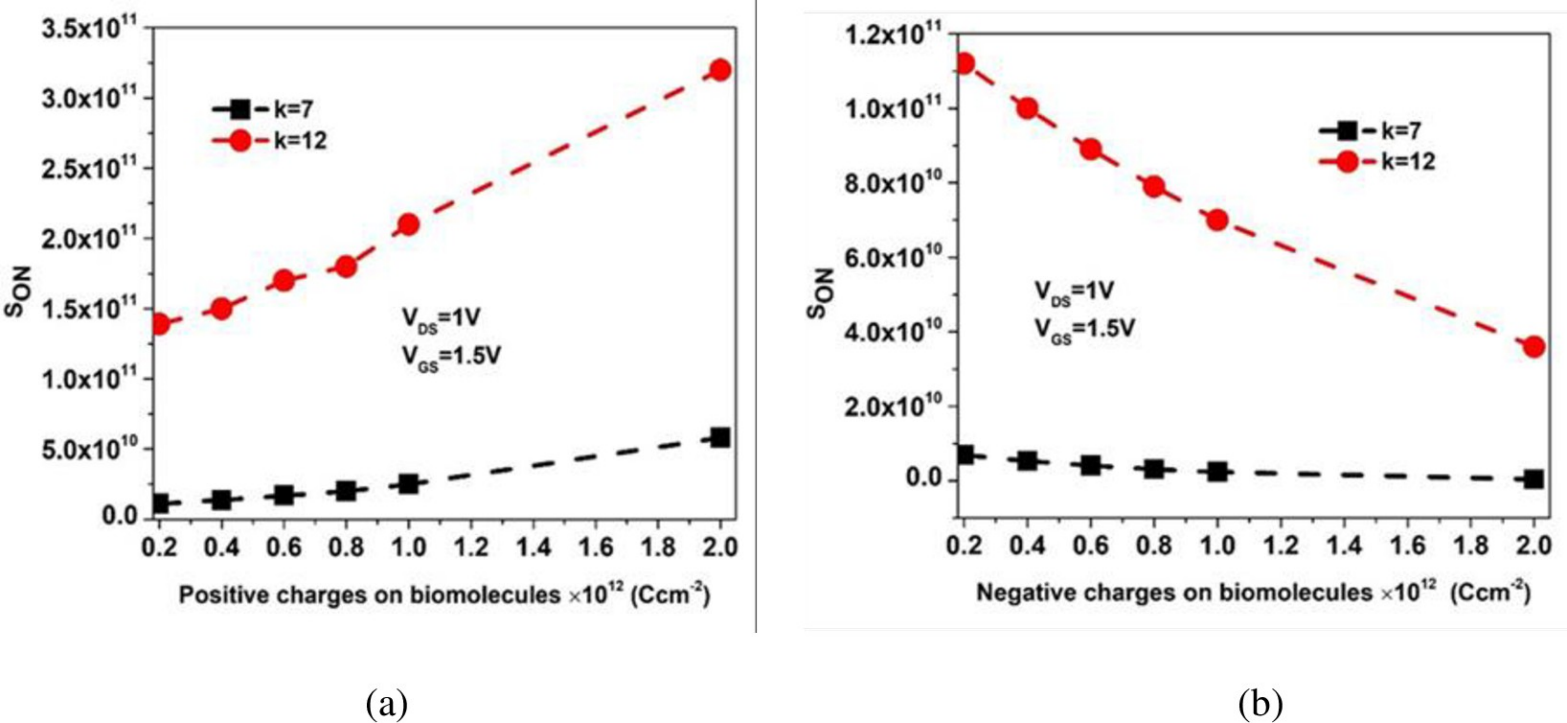
ON current sensitivity of the simulated BM-SO-HTFET biosensor at k = 7, 12 for (a) positive charge (b) negative charged biomolecules.

The relation between the gate voltage and the biomolecule charge density (N) given in (4) represents the field effect transistor voltage balance equation. Where, **Ψ**_***s***_ represents surface potential, N represents biomolecules charge density, C_eff_ represents the effective capacitance per unit area, ***ϕ***_***ms***_ represents the metal-semiconductor work function. There will be increased **Ψ**_***s***_ as +N increments at fixed gate voltage (V_GS_) as shown in (4). This will result in increased sensitivity of BM-SO-HTFET biosensor [21]. Increment in sensitivity takes place as the $\frac{+qN}{C_{eff}}$ component at HfO_2_ increases depleting the intrinsic channel resulting in acute band bending at the tunnelling junction [33]. On the other hand, as -N increases, **Ψ**_***s***_ reduces at fixed V_GS_ that results in hindered device sensitivity. $\frac{-qN}{C_{eff}}$ component at HfO_2_ increases thereby boosting the threshold voltage by broadening the tunnelling span [35,36]. In our work, the biomolecules charge density varies from N = ±**2**×**10**^**11**^ **Ccm**^−**2**^ to N = ±**2**×**10**^**12**^ **Ccm**^**−2**^. Fig 9A and 9B show the sensitivity for positively and negatively charged biomolecules having dielectric constant (k = 7, 12). Maximum sensitivity of ON current reported for positively charged biomolecule is 3.2 × 10^11^ for gelatin biomolecule (k = 12).

### 2.4. Impact of non-ideal issues on S_ON_

**Fig 10:**
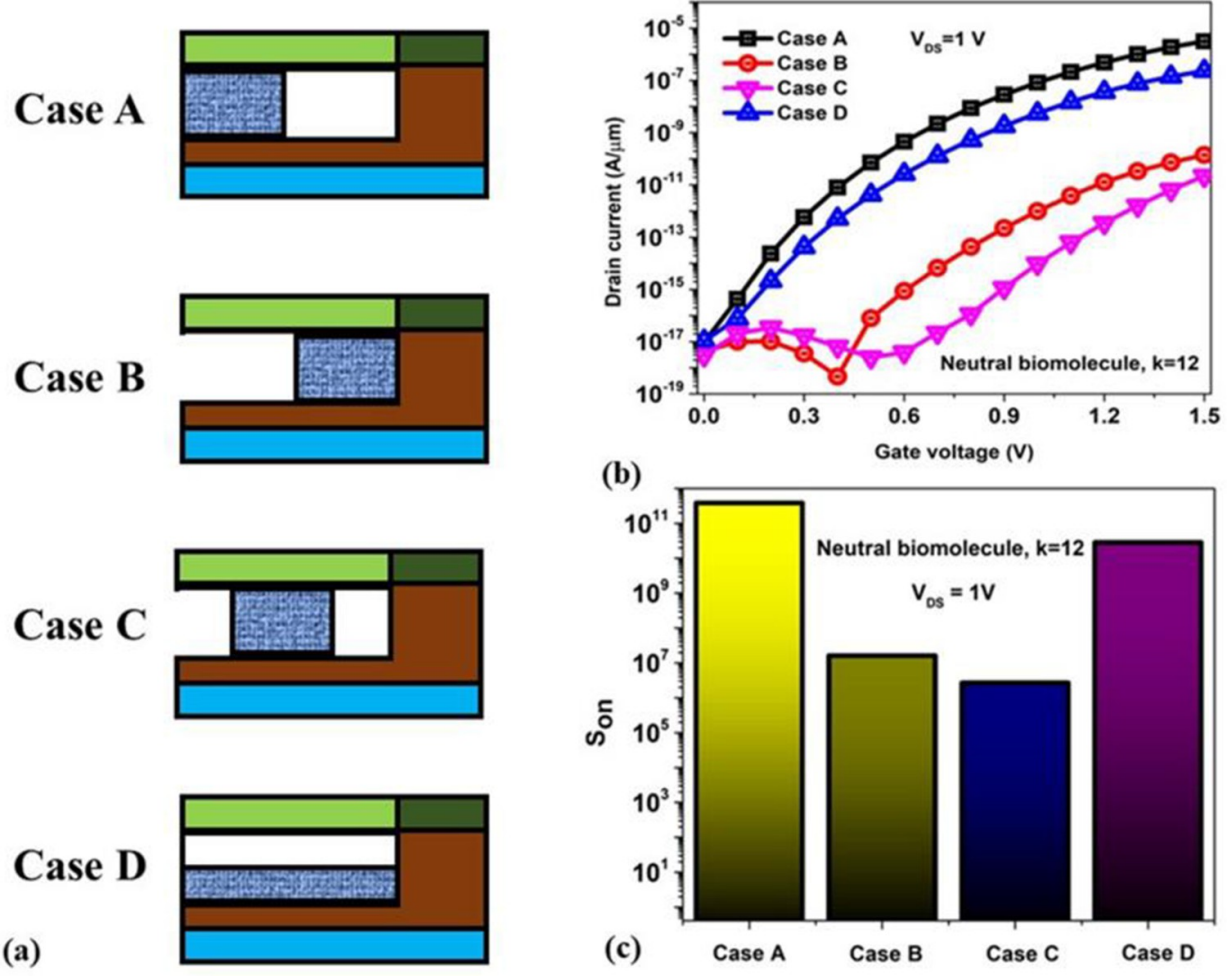
(a) 2D illustration of BM-SO-HTFET biosensor with four different cases of gelatin fill factor (b) transfer characteristics of four different cases (c) ON current sensitivity for four different cases of gelatin fill factor.

This section broadly discusses the real-time manifestation of the biomolecule localization inside the engineered nanogap of the BM-SO-HTFET biosensor, drifting from the ideal case of fully filled nanogap [30,37]. Fig 10A shows four different cases of gelatin (k = 12) localization inside the nanogap with fill factor (β) equal to 50%. Case A, B, C and D determine gelatin with β = 50% at the source-channel tunnelling juncture, channel-drain juncture, mid-nanogap and top air-filled nanogap respectively. It is observed from Fig 10B shows the device transfer characteristics for all the four cases. It is noted that Case A exhibits the highest ON current and almost provides similar behaviour for a β = 100% filled nanogap because of the presence of gelatin at the source-channel tunnelling juncture that facilitates utmost BTBT thereby increased band bending resulting in enhanced ON current. However, for both Case B and Case C it is observed that low ON current is obtained since gelatin is located away from the tunnelling juncture which reduces the gate control and thus hinders BTBT at the same. Even though there is 50% air filled from the top in Case D, the complete localization of gelatin from the tunnelling juncture to the channel-drain interface leads to increased ON current than Case B and Case C. However, the air fill in Case D reduces its BTBT when compared to Case A. Subsequently, the ON current sensitivity is evaluated in Fig 10C. A maximum S_ON_ = 3.8 × 10^11^ is obtained at gelatin with β = 50% for Case A and a minimum S_ON_ = 2.66 × 10^6^ for Case C. Thus, it is understood that in real-time the presence of biomolecules at the source-channel tunnelling juncture even at β = 50% can provide optimum sensing performance.

**Fig 11:**
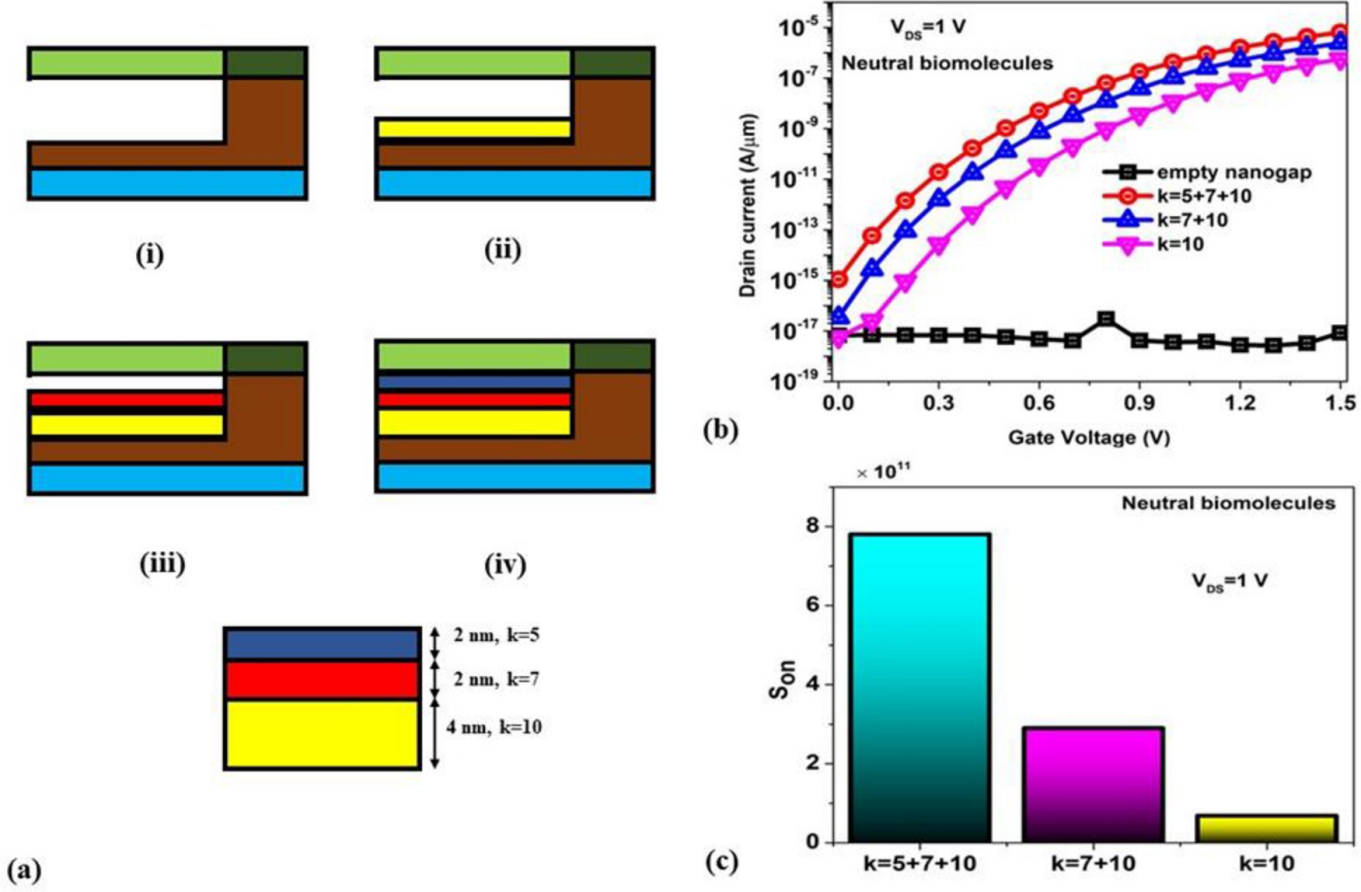
(a) 2D illustrative of BM-SO-HTFET biosensor with (i) air-filled (ii) 4 nm thick keratin filled (iii) 6 nm thick zenin+keratin filled (iv) 8 nm thick gluten+zenin+keratin filled nanogap (b) transfer characteristics of four different cases (c) ON current sensitivity for three stacking cases.

Furthermore, the real-time biosensor performance is even widely investigated by stacking the biomolecules inside the engineered nanogap [37] of the BM-SO-HTFET to determine gluten (k = 5) in Fig 11 for β = 100%. From Fig 11A (i), an empty nanogap (air filled) is considered, (ii) a 4 nm thick keratin (k = 10) is bounded with the nanogap, (iii) a 2 nm thick zenin (k = 7) is stacked on the 4 nm thick keratin, (iv) another 2 nm thick gluten (k = 5) is stacked over the 2nm thick zenin and 4 nm thick keratin for β = 100% nanogap. From the transfer characteristics in Fig 11B, it is observed that the completely stacked biomolecules (k = 5+7+10) inside the nanogap provides maximum ON current and thereby optimum ON current sensitivity of 7.8 × 10^11^ as shown in Fig 11C. Table 2 shows the benchmarking of the BM-SO-HTFET biosensor considering its S_ON_ performance with the existing FET based biosensors. It is reported that the BM-SO-HTFET biosensor exhibits improved ON current sensitivity for neutral biomolecules than almost all the existing biosensors reported in the literature.

**Table 2:** Performance standardization of BM-SO-HTFET biosensor considering current sensitivity.

S.No	FET Based Biosensors	ON Current Sensitivity	
1.	**This work**		4.1 × 10^10^
2.	Biswas, A, et.al **[2022] [34]**		8 × 10^5^
3.	Ghosh R, et.al **[2022] [38]**		8.11 × 10^9^
4.	Reddy N. N, et.al **[2022] [16]**		6.15 × 10^3^
5.	Vanlalawmpuia K, et. al **[2021] [3]**		3.61 × 10^8^
6.	Vanlalawmpuia K, et. al **[2021] [3]**		2.48 × 10^7^
7.	Mukhopadhyay S, et.al **[2020][2]**		1.7 × 10^8^
8.	Wangkheirakpam V. D, et. al **[2020] [20]**		2 × 10^6^
9.	Dwivedi P, et.al **[2020] [39]**		1.05 × 10^2^
10.	Anand, S, et. al **[2019] [40]**		1 × 10^10^
11.	Narang, R, et. al **[2018] [19]**		~0.6
12.	Kanungo S, et. al **[2016] [1]**		1 × 10^5^
13.	Kanungo S, et. al **[2015] [41]**		3 × 10^6^
14.	Cao W, et.al **[2014] [33]**		~10^10^
15.	Szymanski T. H, et.al **[2014] [42]**		9 × 10^3^
16.	Sarkar D, et. al **[2012] [43]**		1 × 10^6^

## 3. Conclusions

In this paper, a high-k stacked gate with bi-metal hetero-juncture TFET is proposed for label free biosensing applications. This study considers the effect of dielectric modulation within the nanocavity beneath the bi-metal gate structure that acts as the sensing site for the biomolecules. The change in dielectric constant (k) and charge densities of the bio-species alter the gate capacitance modulating the device characteristic trend. Hence, the impact of biomolecule immobilization at the binding sites have been studied on band-to-band generation rate, electric field and drain current characteristics of the device. Finally, the ON current sensitivity (S_ON_) of the device is extracted which is utilized as the major sensing metric throughout the work. A better sensitivity (S_ON_ = 3.2 × 10^11^) is reported with Ge content x = 0.4 for positively charged biomolecules in comparison to neutral ones (S_ON_ = 4.1 × 10^10^). The impact of temperature variation depicts a reduction in S_ON_ monotonically with rise in temperature. Moreover, some non-ideal cases are also considered to evaluate the detection limit of the device. From the results, it can be predicted that the reported structure can exhibit optimum sensitivity of S_ON_ = 3.8 × 10^11^ with biomolecules across the source-channel interface even with 50% fill factor. Finally, sensitivity is also determined for stacked biomolecules depicting S_ON_ = 7.8 × 10^11^ for completely stacked biomolecules (k = 5+7+10) inside the nanogap. Comparative analysis of the ON current sensitivity of BM-SO-HTFET biosensor with some of the reported FET based sensors pledges for the potential of the proposed structure for future ultra-sensitive applications.

## Supporting information

S1 Raw data (XLSX)
